# Impact of Beijing healthcare reform on the curative care expenditure of outpatients with noncommunicable diseases based on SHA2011 and interrupted time series analysis

**DOI:** 10.1186/s12913-021-07059-y

**Published:** 2021-10-02

**Authors:** Liming Liu, Yue Xu, Yan Jiang, Liying Zhao, Xuejun Yin, Chen Shen, Yong Yang, Qian Bai, Xiaowei Man, Wei Cheng

**Affiliations:** 1 Beijing University of Chinese Medicine, Beijing, China; 2 The George Institute for Global Health, University of New South Wales, Beijing, China; 3 Institute of Chinese Medical Sciences, University of Macau, Taipa, Macau China; 4 National Institute of Chinese Medicine Development and Strategy, Beijing, China; 5 Beijing, China

**Keywords:** Beijing healthcare reform, Noncommunicable diseases, Curative care expenditure, Outpatient, System of Health Accounts 2011, Interrupted time series analysis

## Abstract

**Background:**

To analyse the changes in curative care expenditure (CCE) associated with noncommunicable diseases (NCDs) before and after the Beijing healthcare reform, thus providing a reference for the healthcare system.

**Methods:**

A total of 60 medical institutions were selected using multistage stratified cluster random sampling in Beijing, China. The records of approximately 100 million outpatients with NCDs in 2016–2018 were extracted. System of Health Accounts 2011 (SHA2011) was used to estimate the CCE. The segmented regression model was established to observe both the instant change and the slope change of intervention in interrupted time series analysis (ITSA). The study was conducted from December 2019 to May 2020 in Beijing, China.

**Results:**

From SHA2011, we found that the CCE for outpatients with NCDs in Beijing were 58.59, 61.46 and 71.96 billion RMB in 2016, 2017 and 2018, respectively. The CCE continued to rise at all hospital levels, namely, tertiary, secondary, and community-level hospitals. However, the proportion of CCE in tertiary hospitals decreased. From ITSA, we can also conclude that the CCE showed a significant increasing trend change at the three hospital levels after the intervention. The drug proportion showed a significant decreasing trend change in secondary and tertiary hospitals.

**Conclusions:**

Beijing healthcare reform does have an impact on the CCE of NCDs.

**Supplementary Information:**

The online version contains supplementary material available at 10.1186/s12913-021-07059-y.

## Background

Over the past decades, China has witnessed great changes in the disease spectrum, with NCDs such as stroke and ischaemic heart disease becoming the top two factors negatively affecting people’s life expectancy [1]. Years of life lost (YLLs), years lived with disability (YLDs), and disability-adjusted life-years (DALYs) due to NCDs have also generally increased [2]. In Beijing city in 2016, it was reported that approximately three-quarters of deaths were caused by three NCDs: malignant tumours, heart disease and cerebrovascular diseases [3]. NCDs have also imposed a heavy economic burden on the healthcare system in China. According to the 2013 Global Burden of Disease Survey, approximately 77 % of national health expenditures were attributed to chronic diseases [4]. Few studies have discussed the medical expenditure of NCDs, which is increasing rapidly [5].

Before the healthcare price reform, the aim of government-dominated price regulation was to set prices for basic health services in public hospitals, including services with large labour inputs and high-tech diagnostic tests, which were usually far below costs. To save public hospitals from financial risk, the government would compensate hospitals and allow them to add a 15 % mark-up on drug sales [6]. Since the mid-1980 s, the government has dramatically cut subsidies, and hospitals have had to make full use of the 15 % drug sale mark-up policy to meet their financial needs, which has resulted in distorted supply-side incentives [7]. Since physicians are usually rewarded according to the monetary values of the drugs they prescribe, they tend to prescribe expensive drugs and to overprescribe drugs to acquire revenue for themselves and their hospitals [8], which is one of the reasons for the rapid growth of health expenditures in recent years [9]. Drug expenditure has been a major source of hospital profit over the long term [10], accounting for 45.5 % of the outpatient medical expenses in 2015 [11]. However, the public has continually complained about high medical costs, especially patients with NCDs who need long-term medication [12].

To abolish the 15 % drug sale mark-up and change the distorted healthcare-providing incentives for physicians and hospitals, the zero-mark-up drug policy was put forward in the 2009 China health reforms. Beijing implemented its new price regulations on April 8, 2017 [13], and mark-ups on drug sales have been disallowed since then (except for herbal medicines) [14]. The prices of hundreds of health service items were also adjusted in this reform, especially those items that require large labour inputs. For example, prices for high-tech diagnostic tests decreased, while outpatient registration and consultation fees were significantly increased. More than 3600 medical establishments were covered in this reform [15].

Current research has confirmed that the burden of medicines has been reduced since the reform [16], and patients now tend to choose community-level hospitals rather than high-level hospitals [17]. A study analysed the immediate and long-term trend changes of the service utilization and payment of different medical insurance groups through interrupted time series analysis. Previous research has mostly shown the influence of the reform on all diseases or specific diseases by observing changes in medical expenses and patient visits [10, 18]. Previous studies were mostly conducted in single medical institutions, the sample sizes were small, and each study’s representativeness was therefore limited. Meanwhile, few studies have addressed whether the healthcare price reform in Beijing significantly reduced the financial burden of patients with NCDs, who ought to benefit from this important reform. Therefore, for the first time, our study observed the effect of the reform on CCE for NCDs of outpatients using the System of Health Accounts 2011. This research included 60 hospitals with different levels and specialties – the representativeness of which is much higher. Thus, this study is an important supplement to the existing literature.

## Methods

### Data source

The health expenditure data (outpatient income, outpatient visits, outpatient project subsidy) were collected from the Beijing Health Statistics Yearbook 2017–2019, the Beijing Health Finance Statistical Yearbook 2017–2019, the Beijing Statistical Yearbook 2017–2019, the China National Health Accounts Report 2017–2019 and the Beijing Health Accounts Report, which are used to account for the CCE. In addition, medical records data (treatment fees, drug and consumables fees, level of institution, main disease diagnosis, and exit-hospital date) were obtained from medical and public health institutions and were used to account for the CCE and to perform the ITSA.

### Sample institutions

The sample institutions include municipal-, district-, and community-level hospitals. Municipal hospitals are under the management of the Beijing Hospital Administration. District- and community-level hospitals are under the management of the health commissions of districts in Beijing.

Municipal-level hospitals: Because of the great difference among municipal-level hospitals in Beijing, most municipal hospitals (19 of 22) were included in the sample institutions. In addition, 5 municipal hospitals that had implemented the zero-mark-up drug policy before April 2017 were excluded from this study. Then, 14 municipal medical institutions were selected.

District- and community-level hospitals: A multistage stratified cluster sampling survey was used. In the first stage, principal component analysis (PCA) was used with five indicators (financial subsidy income, number of health technicians, per capita GDP, per capita government health expenditure, and permanent population density), and the Dongcheng, Fengtai, Changping and Pinggu Districts were selected. In the second stage, streets were randomly selected at a rate of 20 % in each district. In the third stage, 1 hospital was randomly selected in each district, and 1 community hospital was randomly selected on each street. Then, 11 Dongcheng District medical institutions, 12 Fengtai District medical institutions, 12 Changping District medical institutions and 11 Pinggu District medical institutions were selected.

Finally, a total of 60 medical institutions were selected from the sample. Institutions are divided into community hospitals (first level), secondary hospitals (second level) and tertiary hospitals (third level) according to their functionality, size and specialization [19]. The community hospital provides healthcare services for common, frequently occurring and chronic diseases. Secondary hospitals provide comprehensive medical and health services to multiple community hospitals. In addition, tertiary hospitals provide high-level, specialized medical and health services nationwide to treat intractable diseases.

The main diagnosis of the disease was coded using the International Statistical Classification of Diseases and Related Health Problems, 10th Vision (ICD-10). According to the classification of the global burden of disease (GBD), all medical records of outpatients with NCDs in which GBD was coded 2 were extracted. Ultimately, a total of 116,149,047 records were collected in this study. The indicators were summarized by month of exit-hospital date, including CCE and proportion of drug fees out of medical expenditure (drug proportion). Medical expenditures are composed of treatment fees, drug fees and consumables fees.

### Statistical method

#### System of Health Accounts 2011

The “System of Health Accounts 2011” (SHA2011) is universally acknowledged as a method of total health expenditure accounting and was developed by the World Health Organization. This method is suitable for cross analysis of regional health expenditure, including source, institution and service utilization [20]. SHA2011 abandons the expression Total Health Expenditure (THE) but recommends the use of the expression Current Health Expenditure (CHE). Thus, only services that lead to goods consumption are included in the calculation, including medical income, basic expenditure subsidies and special subsidies for government-designated public health projects [21]. After the expenditure for prevention was removed from the CHE, the CCE was incurred [22].

In this study, SHA2011 was used to calculate the CCE of outpatients with NCDs in different institutional levels in Beijing; only curative service data were calculated, while data related to prevention were not included.

Outpatient curative expenditure (${E}_{OCS}$) contains outpatient curative income (${E}_{\text{O}\text{C}\text{I}}$), outpatient project subsidy (${E}_{\text{O}\text{P}\text{S}}$) and outpatient basic curative expenditure subsidy (${E}_{\text{O}\text{B}\text{S}}$). ${E}_{OCI}$ represents direct medical health expenditures, including treatment fees, drug fees and consumable fees.${E}_{OPS}$ is the subsidy provided by finance to develop the construction of clinical specialties and to undertake the planned public health tasks. ${E}_{OBS}$ is the subsidy from finance and mainly includes personnel funds, public funds, and security provisions for curative services, which ensures the daily functioning of hospitals [23]. Outpatient expenditure for prevention has been removed. For more details on the calculation steps and formulas, please refer to the published literature [21].

#### Interrupted time series analysis

Interrupted time series analysis (ITSA) is a quasi-experimental study designed to evaluate the effect of intervention. It is especially suitable for cases where randomized controlled trials are not available, such as policy evaluation [24]. It measures the level effect and the trend effect of an intervention. The level effect refers to the instant change in the intervention, and the trend effect refers to the slope change before and after the intervention. Based on a segmented regression model [25], the level effect and trend effect were both used to evaluate the effect of the intervention. This evaluation requires at least 8 time periods before and after intervention time nodes [26].

In our study, a series of monthly measurements (the CCE and drug proportion) between January 2016 and December 2018 was used to measure the effect of policy intervention. The 15-month preintervention period was defined as January 2016 to March 2017, and the 21-month postintervention period was defined as April 2017 to December 2018. First, the segmented regression model was y = β_0_ + β _1_ · X_1_ + β _2_ · x_2_ + β _3_ · X_3_ + ε _t_, which was employed to fit the standard model in the ITSA analysis. Y is the dependent variable, i.e., the curative expenditure; β _0_ is a constant term, which is the level of Y in the initial stage; X_1_ is the time series, coded as “1, 2, 3…”, which corresponds to the observation points in turn; x_2_ represents the intervention stage of the observation point, with the code “0” before the intervention and “1” after the intervention; X_3_ represents the time series after the intervention, with the observation points before the intervention represented by the code “0”, and the observation points after the intervention represented by the code “1, 2, 3…”. β _1_ is the slope before the intervention; β _2_ is the level change during the intervention; β _3_ is the slope change before and after the intervention; β_1_ + β _3_ is the slope after the intervention; and ε _t_ is the random error. Then, the plot of residuals and the partial autocorrelation were used to test the autocorrelation of dependent variables. Finally, seasonality was included to adjust the model. For more details on the model operation, please refer to the published literature [27]. The ITSA was implemented using R version 3.6.3 (Copyright (c) 2020 the R foundation for statistical computing).

## Results

### Basic results in CCE for noncommunicable diseases

Table 1 shows that in the three hospital levels in Beijing, the CCEs for NCDs were 58.58 billion RMB in 2016, 61.45 billion RMB in 2017 and 71.95 billion RMB in 2018. In all three hospital levels, the CCE increased yearly according to all of the positive growth rates. The fastest growth appeared in community hospitals in 2017 (19.21 %) and in secondary hospitals in 2018 (44.15 %). The growth rate in tertiary hospitals in 2017 and 2018 (2.01 %, 115.55 %) was slower than the overall growth rate (7.16 %, 17.89 %).

**Table 1:** Amount of outpatient curative care expenditure (CCE) for noncommunicable diseases in different levels of medical institutions from 2016 to 2018

Medical institution	2016		2017		2018	
	CCE (billion RMB)	Growth rate (%)	CCE (billion RMB)	Growth rate (%)	CCE (billion RMB)	Growth rate (%)
Community hospital	12.47	-	14.55	19.21	15.74	8.98
Secondary hospital	7.52	-	8.36	13.57	11.97	44.15
Tertiary hospital	38.60	-	38.55	2.01	44.24	15.55
**Total**	**58.58**	**-**	**61.45**	**7.16**	**71.95**	**17.89**

The growth rate is calculated at a comparable price

### Allocation of CCE in different medical institutions levels

**Fig. 1:**
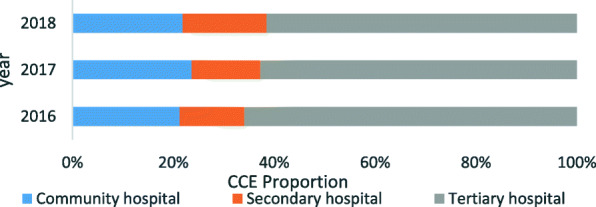
Allocation of the outpatient curative care expenditure (CCE) of noncommunicable diseases in different levels of medical institutions from 2016 to 2018

Figure 1 shows that most CCE for NCDs was spent in tertiary hospitals, which accounted for at least 60 %. In contrast, the ratios of CCE generated in community and secondary hospitals were only approximately 10–25 %. In 2017, the proportion of CCE spent in community hospitals increased by 2.39 %, and the ratio of CCE in secondary hospitals increased by 3.03 % in 2018. The difference in the share of CCE between these kinds of hospitals narrowed from 2016 to 2018.

### Interrupted time series analysis for noncommunicable disease

**Fig. 2:**
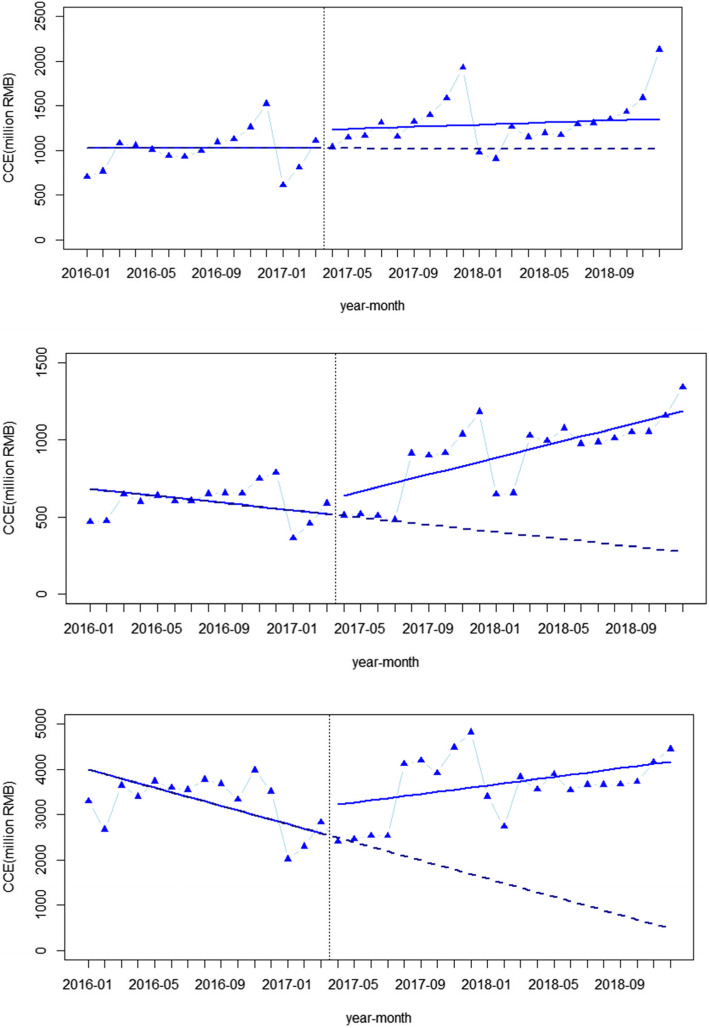
Segmented regression analysis of the outpatient curative care expenditure (million RMB) for noncommunicable diseases in different levels of medical institutions

Figure 2 shows that policy intervention has a significant influence on outpatient CCE for NCDs, and the consequences varied in community, secondary and tertiary hospitals. In community hospitals, the CCE showed a decreasing trend before intervention (*p* < 0.05), and at the moment of intervention. there was a significant increase (*p* < 0.001). After the intervention, the CCE had a trend change of increasing (*p* < 0.001), and the trend of CCE was reversed. In secondary hospitals, CCE decreased (*p* > 0.05) before the intervention, while at the moment of intervention, the CCE experienced a sudden increase (*p* > 0.05). In the after-intervention period, the CCE in secondary hospitals experienced a significant growth trend change (*p* < 0.01). The effects of intervention on CCE in tertiary hospitals were somewhat similar to those in secondary hospitals. Before intervention, the CCE decreased (*p* > 0.05), and at the moment of intervention, the CCE suddenly dropped (*p* > 0.05). After the intervention, it showed a significant growth trend change (*p* < 0.05), and the downward trend was reversed. Seasonality was significant in all three models (*p* < 0.001).

**Fig. 3:**
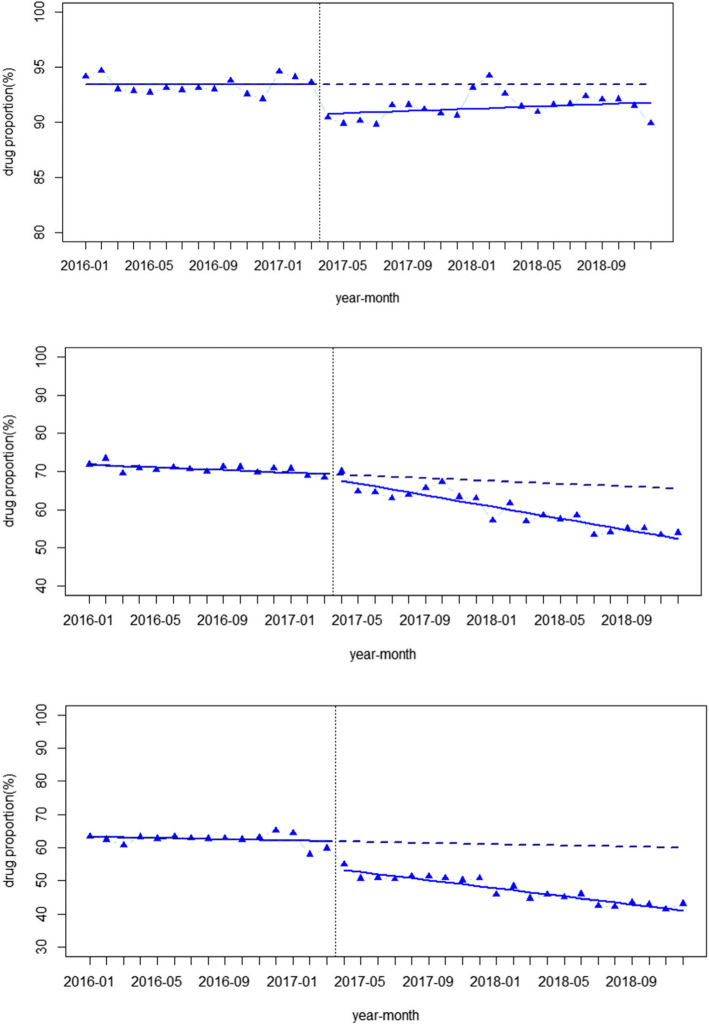
Segmented regression analysis of outpatient drug proportions (%) for noncommunicable diseases in different levels of medical institutions

Figure 3 displays the impact of price reform on drug proportions for NCDs in different hospital levels. In community hospitals, the drug proportion showed an increasing trend before the intervention (*p* > 0.05), while at the time of the intervention, a sudden decline occurred (*p* < 0.001). In the after-intervention period, the drug proportion experienced a trend change of increasing (*p* > 0.05). In secondary hospitals, the drug proportion decreased before the intervention (*p* < 0.001), and at the moment of the intervention, it dropped (*p* > 0.05). Conversely, after the intervention, it showed a decreasing trend change (*p* < 0.001), and the downtrend of the drug proportion increased. The situation in tertiary hospitals is very similar to that in secondary hospitals. Before the intervention, the drug proportion decreased (*p* > 0.05), while at the time of the intervention, it declined suddenly (*p* < 0.001). After the intervention, the downtrend accelerated (*p* < 0.001). Seasonality was significant in all three models (*p* < 0.01).

## Discussion

To our best knowledge, this is the first study to explore the impact of Beijing healthcare price reform on the CCE for outpatients with NCDs using the ITSA results. Overall, the price reform had a significant effect on outpatient CCE, especially in secondary and tertiary hospitals in Beijing city.

The SHA2011 results showed that the CCE increased in all three hospital levels from 2016 to 2018, while the ITSA results suggested that after the drug sale markup was cancelled, the trend of the CCE significantly increased in all three hospital levels. Both of the above results indicated that the amount of CCE for outpatients with NCDs increased after the price reform. The reason may be that when the cost of drugs in tertiary hospitals was lower, expenses for some other medical items increased, and medical service fees were established in the meantime, which was consistent with the price reform measures that adjusted the prices of some medical services to rise while the prices of other medical services fell [28]. In addition, the increase of the CCE in tertiary hospitals may be due to the increase in the amount of visits or the increase in the average cost per visit. According to a previous study, the outpatient visits in tertiary hospitals decreased after the drug markup was cancelled [29]. However, the results of changes in the average cost per visit in current studies were inconsistent, some of which increased and some of which decreased [30–32]. Thus, we can only consider that as the number of outpatients with NCDs was not decreasing, the CCE spent in tertiary hospitals was still increasing. However, according to the SHA2011 results, the growth rates in tertiary hospitals in 2017 and 2018 were slower than the overall growth rate, which is a good sign.

The SHA2011 results showed that the proportion of CCE for NCDs in tertiary hospitals accounted for a large part of the CCE, which was consistent with the distribution of medical expenses in China. This may be related to certain characteristics of Chinese medical system. Tertiary hospitals are large in scale and advanced in technology, and the whole medical level is significantly higher than that in community hospitals; therefore, people tend to choose tertiary hospitals for treatment, especially patients who have more complicated conditions [33]. Therefore, for a long time, tertiary hospitals contributed much higher health expenditures than primary hospitals in China.

The SHA2011 results also revealed that the proportion of CCE for outpatients with NCDs in tertiary hospitals declined for two consecutive years after the reform, which was a positive signal that the CCE dispersed from tertiary to secondary and community hospitals after the drug markup was cancelled, which was consistent with the policy expectations.

In addition, the SHA2011 results demonstrated that the proportion of the CCE in community hospitals increased in the reform year of 2017 and then decreased in 2018 for the following possible reason. As the reform measures proposed, doctors in community hospitals can provide long-term prescriptions for patients with four types of chronic diseases: hypertension, diabetes, coronary heart disease, and cerebrovascular disease. Therefore, doctors may prescribe larger amounts of medicine for patients with NCDs. In addition, as medical service fees were established in the reform, medical service fees in community hospitals were less expensive than those in tertiary hospitals. Thus, while many patients may have preferred to go to the community hospital at the beginning of reform, due to the lack of service capabilities in community hospitals, some patients may have returned to secondary or tertiary hospitals, which has been demonstrated previously [34].

However, the ITSA results revealed that in community hospitals, although drug proportions decreased at the time of the price reform, the trend change did not seem to be significant, which may be related to the cancellation of some common drug markups earlier in community hospitals in 2006 [35]. Studies have shown that after cancelling drug markups in the 2006 reform, the average cost and the average drug cost decreased significantly [36]. Some studies have suggested that the burden of medical care for patients in community hospitals did not change much after the reform, a contributing reason for which may involve the insufficiency of drugs in community hospitals [34]. Since the 2017 reform, the variety of drugs covered by markup cancelling has been expanded, and the range of reimbursement for medicines in the three hospital levels was unified [37]. However, due to the inadequate drug types and poor medical service abilities in community hospitals, patients may still be willing to choose tertiary hospitals instead of community hospitals. In addition, there were significant growth trends of the CCE in secondary and tertiary hospitals after the price reform, as shown in several previous studies [38]. We can explain this phenomenon from both the demand and the supply side. From the demand side, a decline in drug prices may cause an increase in the medical demand of patients; thus the overall medical expenses increase. From the supply side, more Chinese Herbal Medicine, which still has a price markup, as allowed in the policy, might be used. The above reasons are possible factors for the increase of the CCE in secondary and tertiary hospitals.

According to the above factors, the change in the CCE in community hospitals seemed slightly different from the SHA2011 and ITSA results, which may be related to the characteristics of the two methods. The results of SHA2011 illustrated the changes in the year of reform (2017) and the changes in the second year of reform (2018). The annual increase in CCE can intuitively reflect the changes of each year in the SHA2011 results. Conversely, the results of ITSA showed the instantaneous changes (level change) in the month of reform and the long-term trend changes (trend change) after the reform. Moreover, statistical tests are used to determine whether changes are significant, which is why the two results have different emphases.

In addition, the trends of drug proportions decreased significantly in secondary and tertiary hospitals. After the drug markups were cancelled, the prices of drugs dropped. Additionally, doctors could not obtain profits from prescribing more drugs; thus, they no longer prescribed large amounts of drugs. Both the lower price and the decreased usage of drugs bring about the decrease of the drug proportion in the total medical expenses. We may conclude that the reform resulted in a positive effect on the irrational use of drugs. It has been confirmed that reform measures may influence physician profits and further influence physician behaviour [39]. Our findings seem to provide evidence of curative expenditure for NCDs. Although the rational use of drugs can reduce the waste of medical resources, there might be some negative effects, as it was demonstrated in a previous study that the enthusiasm of physicians may decrease. In addition, medical service fees need to be paid for each visit. Therefore, patients were asked to register repeatedly to increase the income from medical service fees. Furthermore, doctors may prescribe more items other than medicines for profit, such as laboratory tests or surgery fees. Therefore, we recommend that comprehensive measures should be taken to rationalize physician behaviour and to reduce excessive medical costs, such as increasing prices to reflect the true value of technical services to motivate doctors.

## Conclusions

Beijing healthcare reform seems to have had a comprehensive effect on the CCE of NCDs. As a whole, this reform has had an effective impact on the burden of drugs, especially in secondary and tertiary hospitals. Moreover, SHA2011 can provide more macro and representative data for health systems. This study is an important supplement to previous studies that assessed the influence of public hospital reform on institutions at different levels.

## Supplementary Information

**Additional file 1.** The survey guide.

## Data Availability

All data generated or analysed during this study are included in this published article.
