# Supplementary material for: Impact of Beijing healthcare reform on the curative care expenditure of outpatients with noncommunicable diseases based on SHA2011 and interrupted time series analysis
Source: BMC Health Serv Res. 2021 Oct 2;21:1045. doi: 10.1186/s12913-021-07059-y (PMC8487539; doi:10.1186/s12913-021-07059-y)
Supplement: Supplementary file 1 — Additional file 1. The survey guide. [file 12913_2021_7059_MOESM1_ESM.docx]

**The survey guide**

**1.Introduction of SHA2011**

The survey was based on the framework of "System of Health Accounts 2011" (SHA2011), which is an international tool for health financing analysis and evaluation. It is suitable for cross analysis of regional health expenditure, including source, institution and service utilization. Thus, it can show the basic results and the distributions of curative care expenditure (CCE). Only services that lead to goods consumption are included in the calculation, including medical income, basic expenditure subsidy and special subsidy for government-designated public health projects.

**2.The sample institutions**

The sample institutions include municipal-, district-, and community-level hospitals in Beijing.

Municipal-level hospitals: Because of the great difference among municipal-level hospitals in Beijing, most municipal hospitals (19 of 22) were included in the sample institutions. In addition, 5 municipal hospitals that had implemented the zero-mark-up drug policy before April 2017 were excluded from this study.

District- and community-level hospitals: A multistage stratified cluster sampling survey was used. In the first stage, principal component analysis (PCA) was used with five indicators (financial subsidy income, number of health technicians, per capita GDP, per capita government health expenditure, and permanent population density), and the Dongcheng, Fengtai, Changping and Pinggu Districts were selected. In the second stage, streets were randomly selected at a rate of 20% in each district. In the third stage, 1 hospital was randomly selected in each district, and 1 community hospital was randomly selected on each street.

Finally, 11 Dongcheng District medical institutions, 12 Fengtai District medical institutions, 12 Changping District medical institutions and 11 Pinggu District medical institutions were selected from the sample.

**3.The data collection**

*3.1 Basic statistical data*

The health expenditure data (outpatient income, outpatient visits, outpatient project subsidy) were collected from the Beijing Health Statistics Yearbook 2017-2019, the Beijing Health Finance Statistical Yearbook 2017-2019, the Beijing Statistical Yearbook 2017-2019, the China National Health Accounts Report 2017-2019 and the Beijing Health Accounts Report, which are used to account for the CCE. In addition, medical records data (treatment fees, drug and consumables fees, level of institution, main disease diagnosis, and exit-hospital date) were obtained from medical and public health institutions and were used to account for the CCE and to perform the ITSA.

*3.2Field survey data*

The field survey data is used to calculate the ratio of preventive service cost. The manpower input of public health services was collected through the online system. After the data is collected, the integrity and authenticity of the data are reviewed.
